# Morphological and molecular response of small intestine to lactulose and hydrogen-rich water in female piglets fed *Fusarium* mycotoxins contaminated diet

**DOI:** 10.1186/s40104-019-0320-2

**Published:** 2019-02-13

**Authors:** Xu Ji, Qing Zhang, Weijiang Zheng, Wen Yao

**Affiliations:** 1 Laboratory of Gastrointestinal Microbiology, Jiangsu Key Laboratory of Gastrointestinal Nutrition and Animal Health, College of Animal Science and Technology, Nanjing Agricultural University, Nanjing, 210095 Jiangsu China; 2 Key Lab of Animal Physiology and Biochemistry, Ministry of Agriculture, Nanjing, 210095 Jiangsu China

**Keywords:** *Fusarium* mycotoxins, Hydrogen-rich water, Lactulose, Piglets, Small intestine

## Abstract

**Background:**

Following the intake of *Fusarium* mycotoxin-contaminated feed, small intestines may be exposed to high levels of toxic substances that can potentially damage intestinal functions in livestock. It is well known that *Fusarium* mycotoxins will lead a breakdown of the normally impeccable epithelial barrier, resulting in the development of a “leaky” gut. H_2_ administration with different methods has been proved definitely potentials to prevent serious intestinal diseases. The goal of this study is to investigate the roles of lactulose (LAC) and hydrogen-rich water (HRW) in preventing intestinal dysfunction in piglets fed *Fusarium* mycotoxin-contaminated feed.

**Methods:**

A total of 24 female piglets were evenly assigned to 4 groups: negative control (NC) group, mycotoxin-contaminated (MC) feed group, MC feed with LAC treatment (MC + LAC), and MC feed with HRW treatment (MC + HRW), respectively. Piglets in the NC group were fed uncontaminated control diet, while remaining piglets were fed *Fusarium* mycotoxin-contaminated diet. For the NC and MC groups, 10 mL/kg body weight (BW) of hydrogen-free water (HFW) was orally administrated to piglets twice daily; while in the MC + LAC and MC + HRW groups, piglets were treated with the same dose of LAC solution (500 mg/kg BW) and HRW twice daily, respectively. On d 25, serum was collected and used for biochemical analysis. Intestinal tissues were sampled for morphological examination as well as relative genes and protein expression analysis.

**Results:**

Our data showed that *Fusarium* mycotoxins induced higher serum diamine oxidase (DAO) activities (*P <* 0.05), *D*-lactic acid levels (*P <* 0.01), and endotoxin status (*P <* 0.01), lower villus height (*P <* 0.01) and ratio of villus height to crypt depth (*P <* 0.05) in small intestine, greater apoptosis index and higher mRNA expression related to tight junctions (*P <* 0.05). In addition, the distribution and down-regulation of claudin-3 (CLDN3) protein in the small intestinal was also observed. As expected, oral administrations of HRW and LAC were found to remarkably provide beneficial effects against *Fusarium* mycotoxin-induced apoptosis and intestinal leaking. Moreover, either HRW or LAC treatments were also revealed to prevent abnormal intestinal morphological changes, disintegrate tight junctions, and restore the expression and distribution of CLDN3 protein in the small intestinal mucosal layer in female piglets that were fed *Fusarium* mycotoxins contaminated diet.

**Conclusions:**

Our data suggest that orally administrations of HRW and LAC result in less *Fusarium* mycotoxin-induced apoptosis and leak in the small intestine. Either HRW or LAC treatments could prevent the abnormal changes of intestinal morphology and molecular response of tight junctions as well as restore the distribution and expression of CLDN3 protein of small intestinal mucosa layer in female piglets that were fed *Fusarium* mycotoxins contaminated diet.

**Electronic supplementary material:**

The online version of this article (10.1186/s40104-019-0320-2) contains supplementary material, which is available to authorized users.

## Background

Trichothecenes such as deoxynivalenol (DON) and zearalenone (ZEN) are the major *Fusarium* mycotoxins found in maize or feed ingredients contaminated by *Fusarium* fungal [1]. Gastrointestinal tract (GIT) as the first physical barrier protects the body from different kinds of contaminants, is the place where mycotoxins absorption and metabolization occur. Recently, there is increasing attention on the adverse effects of *Fusarium* mycotoxins on the physical structure and integrity of intestines [2, 3]. *Fusarium* mycotoxins contaminated diet has been found to alter intestinal morphology resulting in villus atrophy and reduced villi height [4], reduced nutrition absorption [5, 6], affected the expression of junctional adherent protein and tight-junction protein [7]. In addition, *Fusarium* mycotoxins can also induce inflammation and oxidative stress in intestinal epithelial cells [8], accelerating cell apoptosis, thus affecting intestinal mucosa membrane integrity [9, 10]. Ultimately, those changes in intestines lead to increased intestinal permeability and decreased intestinal physical and molecular function [3]. Therefore, the implementation of practical and affordable ways to reduce or remedy the toxic effects of *Fusarium* mycotoxins on intestinal functions are becoming increasingly important.

Molecular hydrogen (H_2_ or hydrogen gas) has many biological effects in animals, including anti-apoptotic, anti-inflammatory, and anti-oxidant [11, 12]. H_2_ administration with different methods has been proved to prevent serious intestinal diseases [13–15]. Among the different methods of how to ingest molecular hydrogen, hydrogen-rich water/saline is one of the most convenient and safe way. Hydrogen-rich saline administrated via tail vein was shown to ameliorate histologic damage, as well as its ability to inhibit ischemia-reperfusion (I/R)-induced apoptosis and to promote epithelial cell proliferation in rats [16]. Luminal injection of hydrogen-rich solution also suppressed apoptosis and intestinal tissue injury in rat intestinal IR injury model [17].

Physiologically, a huge volume of hydrogen gas is generated daily by bacteria inside the GIT during the fermentation of residual undigested carbohydrates [18]. A part of endogenous hydrogen can be further metabolized by bacteria [19], while most of them could be diffused or absorbed into the bloodstream and transported to other host organs [20]. Therefore, supplementation of hydrogen-producing prebiotic could be a viable solution to provide functional hydrogen to animals. The beneficial effects of endogenous H_2_ produced by fructooligosaccharides, inulin, pectin, resistant starches, turmeric, and lactulose have been well explored [21–24].Bacterial fermentation of lactulose could dramatically increase endogenous hydrogen production, which in turn protect against intestinal damage on the models of trinitrobenzene sulfonic acid [25] and dextran sulfate sodium (DSS) [24, 26].

To the best of our knowledge, no study has been reported to assess the effects of HRW or LAC on *Fusarium* mycotoxin-induced intestinal damage in piglets. In this study, we hypothesized that both HRW and LAC can partly reverse damages caused by *Fusarium* mycotoxin-contaminated diet in female piglets, helping to maintain integrity, morphology, and barrier functions of small intestines.

## Methods

### Experimental diets

*Fusarium* mycotoxins contaminated or uncontaminated corn, and the two experimental diets (NC and MC diet, respectively) were prepared as previously described [27, 28]. Briefly*, Fusarium* mycotoxin-contaminated or uncontaminated (control) corn was used to replace 44.5% of the normal maize in the feed. Additional file 1: Table S1 shows the ingredients of NC and MC diets, respectively. Previously, our data on the compositions of *Fusarium* mycotoxins suggested a higher mycotoxins levels in MC diet than NC diet [27, 28]*.*

### Animals, housing, and experimental design

The experimental design was previously described [27, 28]. Twenty-four female piglets (Landrace × Large × White) from six litters (4 piglets/litter) were used in this study with an initial average body weight of 7.25 ± 1.02 kg. Piglets from each litter were equally assigned into one of the 4 treatment groups and individually housed in pens (1.2 m × 2.0 m) with one feeder and one nipple drinker. The piglets had *ab libitum* access to feed and water. The animal trial consisted of a 6-day adaption period and a 25 d experimental period. Piglets in the NC group received uncontaminated NC diet, while the MC, MC + LAC and MC + HRW groups received *Fusarium* mycotoxin-contaminated (MC) diet.

Oral administrations of four different treatments were also as described in our previous studies [27, 28]. Piglets in each group orally received their corresponding treatment twice daily (10:00 and 14:00 h, respectively). Hydrogen-free water (HFW)(10 mL/kg BW) was orally administrated to both NC and MC groups. The HRW containing 0.6–0.8 mmol/L hydrogen (Beijing Hydrovita Biotechnology Company, Beijing, China) was given to the piglets by gavage in MC + HRW group. Piglets in the MC + LAC group were administrated a dose of 500 mg/kg BW lactulose solution (Duphalac, Abbott Healthcare Products, Weesp, The Netherland) dissolved in 10 mL/kg BW volume of HFW. Due to poor health status, there was one piglet removed from each MC, MC + HRW, and MC + LAC treatments, respectively.

### Sample collection and preparation

On d 24, prior to morning feeding, a blood sample was collected from the anterior vena cava of each piglet. After being placed on ice for 30 min followed by centrifugation at 3,000×*g* for 20 min at 4 °C, serum was harvested and stored in a pyrogen-free glass tube at − 80 °C before analysis. By the end of the experiment, piglets were fasted overnight (12 h) and euthanized by an intramuscular injection of sodium pentobarbital (40 mg/kg BW) after 30 min of treatments. The duodenum (5 cm from the gastric cardia), jejunum (8 cm before the end of jejunal Peyer’s patches) and ileum (8 cm from the ileal-caecal junction) segments were collected separately and fixed in 4% paraformaldehyde for 24 h before histological assays. The inner linings of duodenum, jejunum, and ileum were scraped with a smooth glass coverslip to collect mucosa samples. Samples were stored in liquid nitrogen and then at − 80 °C before RNA isolation and western blot analysis.

### Serum chemical analysis

Serum *D*-lactic acid status and diamine oxidase (DAO) activity were measured by enzymatic spectrophotometry using a commercial kit (Jiancheng Bioengineering Institute of Nanjing, Nanjing, Jiangsu, China). Free lipopolysaccharide (LPS) in the serum was measured by a chromogenic end-point Tachypleus Amebocyte Lysate assay kit (Chinese Horseshoe Crab Reagent Manufactory, Xiamen, China) with a minimum detection limit of 0.01 endotoxin units (EU)/mL.

### Morphological analysis of the small intestine

Following fixation in 4% paraformaldehyde for 24 h, the intestinal segments were embedded in paraffin and 5 μm sections were sectioned with a rotary microtome. Then, the sections were stained with hematoxylin and eosin (H&E) and examined under a light microscope (Olympus, Tokyo, Japan). Photomicrographs were taken with an Olympus CKX31 microscope (Olympus, Tokyo, Japan). The morphometric analysis was performed on 10 randomly-selected, well-oriented villi and crypts per piglet. A computerized microscope-based image analyzer (Olympus dotslide Virtual Slide System, Tokyo, Japan) was used to determine the height of villus (from the tip of the villus to the villus-crypt junction) and crypt depth (from the crypt-villus junction to the base of the crypt).

### Determination of apoptosis by terminal deoxynucleotidyl transferase dUTP nick end labeling (TUNEL)

DNA fragments on paraffin-embedded sections of three small intestine parts (duodenum, jejunum, and ileum) were detected by One Step TUNEL Apoptosis Assay Kit (Beyotime, Nantong, China). Ten randomly selected photos were taken with a laser-scanning confocal microscope (Zeiss LSM 700 META; Jena, Germany). The total numbers of cells undergoing apoptosis were counted. The apoptosis index (AI) was calculated by the equation: AI = number of apoptotic cells / total number of cells × 100%.

### Quantitative gene expression analysis

Total RNA from the duodenum, jejunum, and ileum mucosa were isolated using FastRNA® Pro Green Kit (MP Biomedicals, USA). The yield and purity of mRNA were measured spectrophotometrically (Nanodrop 2000, Thermo Fisher, USA). Reverse transcription was conducted using a Prime Script™ RT reagent Kit with gDNA Eraser (Perfect Real Time) (Takara, Dalian, China). qRT-PCR was performed using SYBR Premix Ex Taq™ (Takara, Dalian, China) with the QuantStudio 5 Real-Time PCR System (Thermo Fisher, USA). Specific gene primers for B-cell CLL/lymphoma 2 (*Bcl-2*), caspase-3 and fas cell surface death receptor (*FAS*), zonula occludens 1 (*ZO-1*), occludin (*OCLN*), claudin-1 (*CLDN1*), and claudin-3 (*CLDN3*) (Additional file 2: Table S2) were detected. The real-time PCR reactions were performed using the following cycle program: precycling at 95 °C for 30 s followed by 40 cycles of denaturization for 5 s at 95 °C and annealing for 30 s at 60 °C. β-actin was used as a reference gene for normalization. The relative mRNA expression levels of the target gene in comparison with the reference gene were calculated using the 2^–ΔΔCt^ method.

### Western blot analysis

Intestinal mucosa samples were lysed using RIPA buffer (Roche, Shanghai, China). The concentrations of protein in samples were measured by the bicinchoninic acid (BCA) protein assay kit (Beyotime, Nantong, China). The total protein samples were separated through a 10% SDS polyacrylamide gel and then transferred to a nitrocellulose membrane (Boster, Wuhan, China). The membrane was incubated in 1:10,000 monoclonal mouse anti-beta actin (Bioworld, USA), and 1:1,000 rabbit polyclonal CLDN3 antibodies (Abcam, Shanghai, China) at 4 °C overnight. Then membrane was incubated in 1:10,000 diluted horseradish peroxidase (HRP)-conjugated anti-rabbit antibody (Bioworld, USA) or 1:10,000 diluted HRP-conjugated anti-mouse antibody (Bioworld, USA) for 1 h at room temperature. Tanon™ High-sig ECL Western Blotting Substrate (Tanon, Shanghai, China) was applied to the membrane for 5 min after secondary antibody incubation. The chemiluminescent signals were visualized by the Versa Doc™ imaging system. Signal intensity was quantified using Quantity One software (Bio-Rad, USA). Protein expression levels were normalized with β-actin expression level.

### Immunohistochemistry

Small intestine parts (duodenum, jejunum, and ileum) were immersed in 4% paraformaldehyde for 24 h, dehydrated in ethanol, and embedded in paraffin. The tissues were sectioned into 5 μm thickness on a rotary microtome. After antigen retrieval in 10 mmol/L citrate buffer (pH 7.5) for 3 min in a microwave, paraffin sections were deparaffinized. Tissue endogenous peroxidase activity was blocked with 30% H_2_O_2_ (Sinopharm Chemical Reagent Co., Ltd., Shanghai, China) in methanol (1 h). Sections were incubated with 3% bovine serum albumin (BSA) (DSBIO, Beijing, China) before overnight incubation with CLDN3 (1:200 diluted) antibody (Abcam, Shanghai, China) at 4 °C. Tissue sections were then incubated with biotinylated goat anti-rabbit secondary antibody (1:100, Boster, Wuhan, China) followed by strept avidin-biotin complex (SABC) (1:100, Boster, Wuhan, China). Diaminobenzidine (DAB) (DSBIO, Beijing, China) solution was used to stain the prepared slides for 5 min. After immunoreaction, the images were captured on each slide at Olympus CKX31 microscope (Olympus, Tokyo, Japan). The average density of positive cells was quantified using the Image-Pro Plus software (Media Cybernetics, Bethesda, MD, USA).

### Statistical analysis

Statistical analysis was performed by the one-way ANOVA procedure of SPSS 18.0 (SPSS, Inc., Chicago, IL, USA, 2009). Differences among treatments were compared using Turkey-Kramer test and considered statistically significant at *P* < 0.05.

## Results

### Status intestinal mucosal permeability

Compared with the NC diet, piglets fed *Fusarium* mycotoxins-contaminated diet (MC) had higher DAO activity (*P* < 0.05), and greater concentrations of *D*-lactic acid and endotoxin (*P* < 0.01) (Table 1). Oral administration of either HRW or LAC lower the DAO activities, *D*-lactic acid levels, and LPS concentrations in MC + LAC and MC + HRW piglets compared with the MC group (*P* < 0.05). No difference was found among the NC, MC + LAC and MC + HRW groups in above-mentioned tests.

**Table 1:** Effects of lactulose and hydrogen-rich water on serum *D*-lactic acid levels, diamine oxidase (DAO) activities and endotoxin concentrations in female piglets fed *Fusarium* mycotoxin-contaminated diet^1, 2^

Item	NC	MC	MC + LAC	MC + HRW	SEM	*P*-value
*D*-Lactic acid, mmol/L	12.53^Bb^	21.47^Aa^	14.15^Bb^	14.14^Bb^	1.04	< 0.001
DAO, U/mL	13.37^b^	19.97^a^	13.52^b^	13.68^b^	0.96	0.020
Endotoxin, EU/L	0.68^Bb^	1.26^Aa^	0.74^Bb^	0.84^ABb^	0.06	0.001

Data are expressed as mean with standard error of mean (SEM)

^(A,B,a,b)^Means in the same row not sharing the same lower (*P* < 0.05) or upper (*P* < 0.01) case superscript letters are significantly different

^1^ NC (negative control), basal diet; MC, *Fusarium* mycotoxin-contaminated diet; MC + LAC, MC diet + lactulose treatment; and MC + HRW, MC diet + hydrogen-rich water treatment

^2^ *n* = 5

### Small intestinal morphological changes

No difference was found on crypt depth in duodenum, jejunum, and ileum among the four groups (Table 2). Compared with the NC group, piglets fed MC diet had a lower height of villus in duodenum, jejunum, and ileum (*P* < 0.01). Compared with the MC group, both HRW and LAC treatments attenuated the reduction of villus height in small intestines (duodenum, jejunum, and ileum) caused by *Fusarium* mycotoxin-contaminated diet (*P* < 0.01). In the duodenum and jejunum, the ratio of villus height to crypt depth in the MC group was lower than the NC group (*P* < 0.01). Compared with the MC group, oral administrations of HRW and LAC increased the value of villus height to crypt depth (*P* < 0.01) in both duodenum and jejunum. And no difference was observed among MC, MC + HRW, and MC + LAC groups for the ratio of villus height to crypt depth in the ileum.

**Table 2:** Effects of lactulose and hydrogen-rich water on small intestinal morphology in female piglets fed *Fusarium* mycotoxin-contaminated diet^1, 2^

Item	NC	MC	MC + LAC	MC + HRW	SEM	*P*-value
Duodenum						
Villus height, μm	386.08^Aa^	313.51^Bb^	366.65^Aa^	360.49^Aa^	6.86	< 0.001
Crypt depth, μm	268.13	281.75	272.35	272.50	3.85	0.678
Villus height: crypt depth ratio	1.45^Aa^	1.11^Bb^	1.36^Aa^	1.32^Aa^	0.03	0.001
Jejunum						
Villus height, μm	390.60^Aa^	322.41^Bb^	383.75^Aa^	380.46^Aa^	7.03	< 0.001
Crypt depth, μm	246.11	240.94	247.31	231.89	2.73	0.172
Villus height: crypt depth ratio	1.59^Aa^	1.34^Bb^	1.55^Aa^	1.64^Aa^	0.03	< 0.001
Ileum						
Villus height, μm	376.88^Aa^	334.76^Bb^	366.28^Aa^	363.09^Aa^	4.41	0.001
Crypt depth, μm	240.35	235.78	240.00	257.36	3.36	0.097
Villus height: crypt depth ratio	1.57^a^	1.43^ab^	1.53^ab^	1.41^b^	0.02	0.030

Data are expressed as mean with standard error of mean (SEM)

^(A,B,a,b)^Means in the same row not sharing the same lower (*P* < 0.05) or upper (*P* < 0.01) case superscript letters are significantly different

^1^ NC (negative control), basal diet; MC, *Fusarium* mycotoxin-contaminated diet; MC + LAC, MC diet + lactulose treatment; and MC + HRW, MC diet + hydrogen-rich water treatment

^2^ *n* = 5

**Fig. 1:**
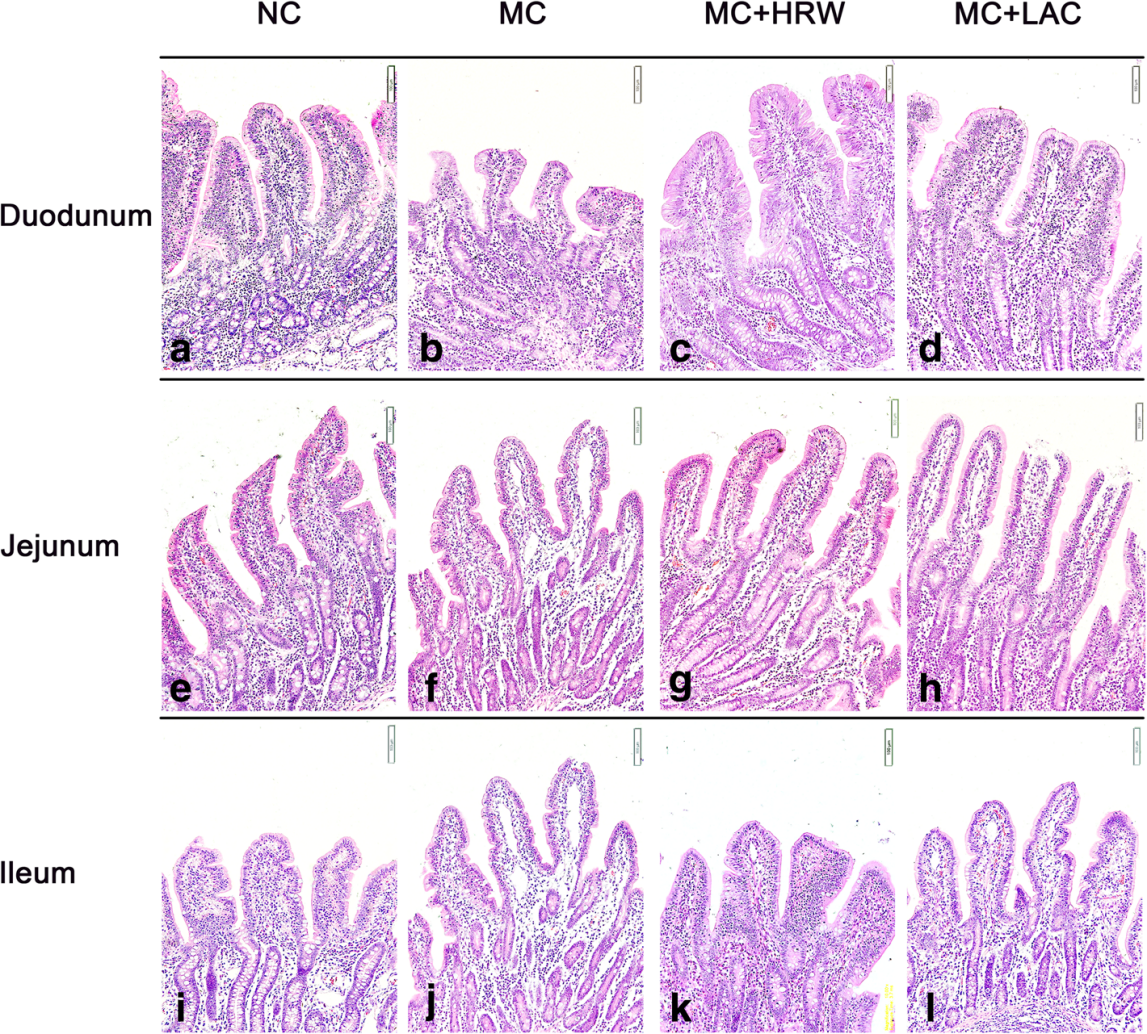
Effects of lactulose and hydrogen-rich water on histological changes of the small intestine in female piglets fed *Fusarium* mycotoxin-contaminated diet. Representative haematoxylin & eosin (H&E) staining images were obtained at 200× magnification with black bar = 100 μm. **a**-**d** duodenum tissue images. **e**-**h** jejunum tissue images. **i**-**l** ileum tissue images. NC (negative control), basal diet; MC, *Fusarium* mycotoxin-contaminated diet; MC + LAC, MC diet + lactulose treatment; and MC + HRW, MC diet + hydrogen-rich water treatment

Representative morphologic observations of the intestinal tissue in the duodenum (Fig. 1a, b, c, and d), jejunum (Fig. 1e, f, g, and h), and ileum (Fig. 1i, j, k, and l) from NC, MC, MC + LAC, and MC + HRW groups are shown in Fig. 1. Morphology examination revealed that obvious denuded to the villi and damages were found in piglets from MC group than NC group. However, these morphological changes in duodenum and ileum were not seen in HRW or LAC groups.

### Small intestinal mRNA changes related to apoptosis genes

**Fig. 2:**
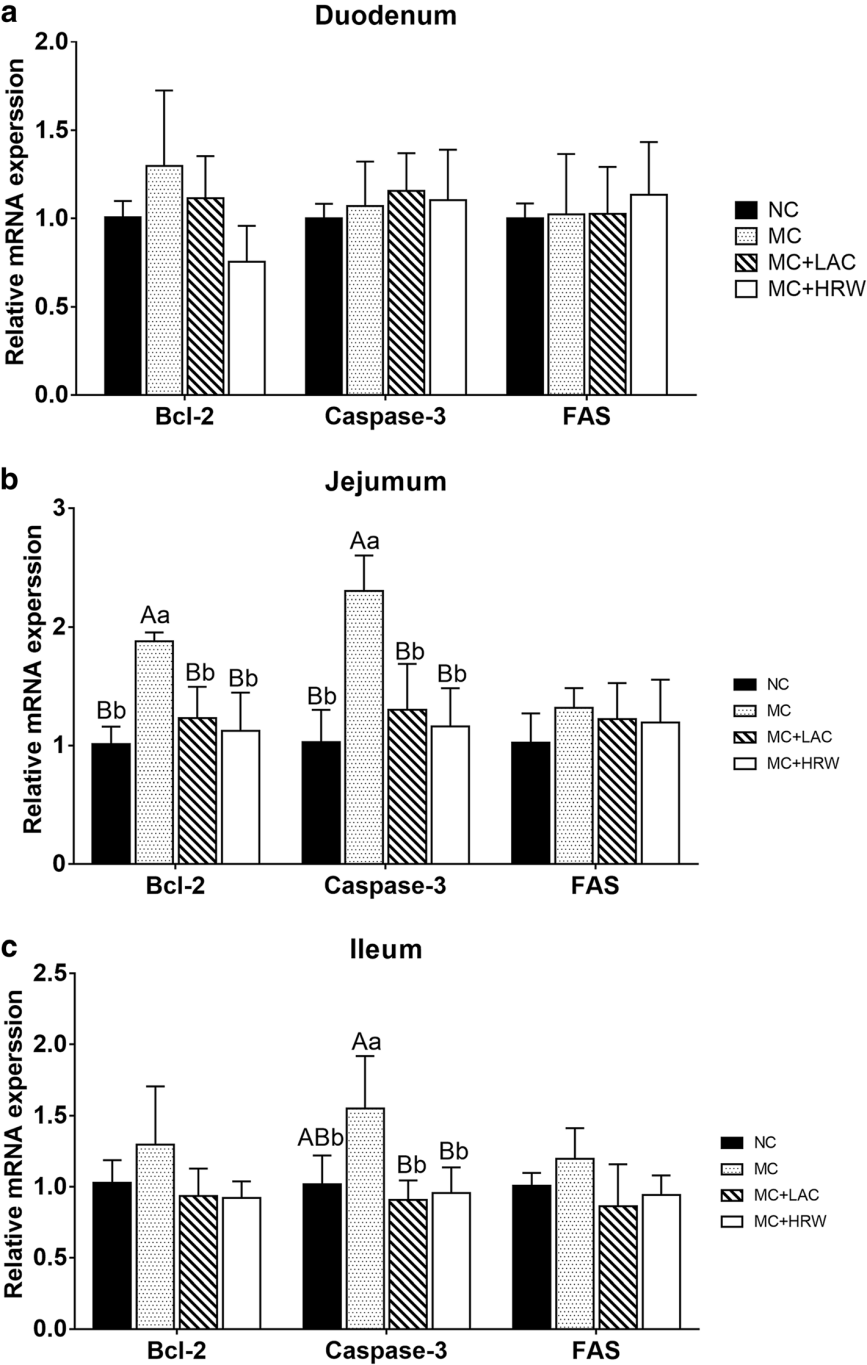
Effects of lactulose and hydrogen-rich water on relative mRNA gene expression levels related to apoptosis in the duodenum (**a**), jejunum (**b**), and ileum (**c**) of female piglets fed *Fusarium* mycotoxin-contaminated diet. Each column represents the mean values (*n* = 5), with their standard deviation (SD) represented by vertical bars. Letters above the bars not sharing the same lower (*P* < 0.05) or upper (*P* < 0.01) case superscript are significantly different. *Bcl-2* = B-cell CLL/lymphoma 2, *FAS* = Fas cell surface death receptor. NC = basal diet; MC = *Fusarium* mycotoxin-contaminated diet; MC + LAC = *Fusarium* mycotoxin-contaminated diet with lactulose treatment. MC + HRW = *Fusarium* mycotoxin-contaminated diet with hydrogen-rich water treatment

No difference was found on mRNA expression levels of *Bcl-2*, caspase-3 and *FAS* in the duodenum among the four groups (Fig. 2a). In jejunum (Fig. 2b), mRNA expression levels of *Bcl-2* and caspase-3 in the MC group were higher (*P* < 0.05) and those in the NC group. However, mRNA expression levels of *Bcl-2* and caspase-3 in the MC + HRW and MC + LAC groups were lower (*P* < 0.01) than those in the MC group. *FAS* mRNA expression levels in jejunum had no difference among the four groups. In ileum (Fig. 2), mRNA expression levels of *Bcl-2* and *FAS* were not different among the four treatment groups. MC group showed higher (*P* < 0.05) caspase-3 gene mRNA expression level than NC group. However, ileum caspase-3 mRNA expression levels were not different among the NC, MC + HRW and MC + LAC groups.

### Intestinal apoptosis status detected by TUNEL

**Fig. 3:**
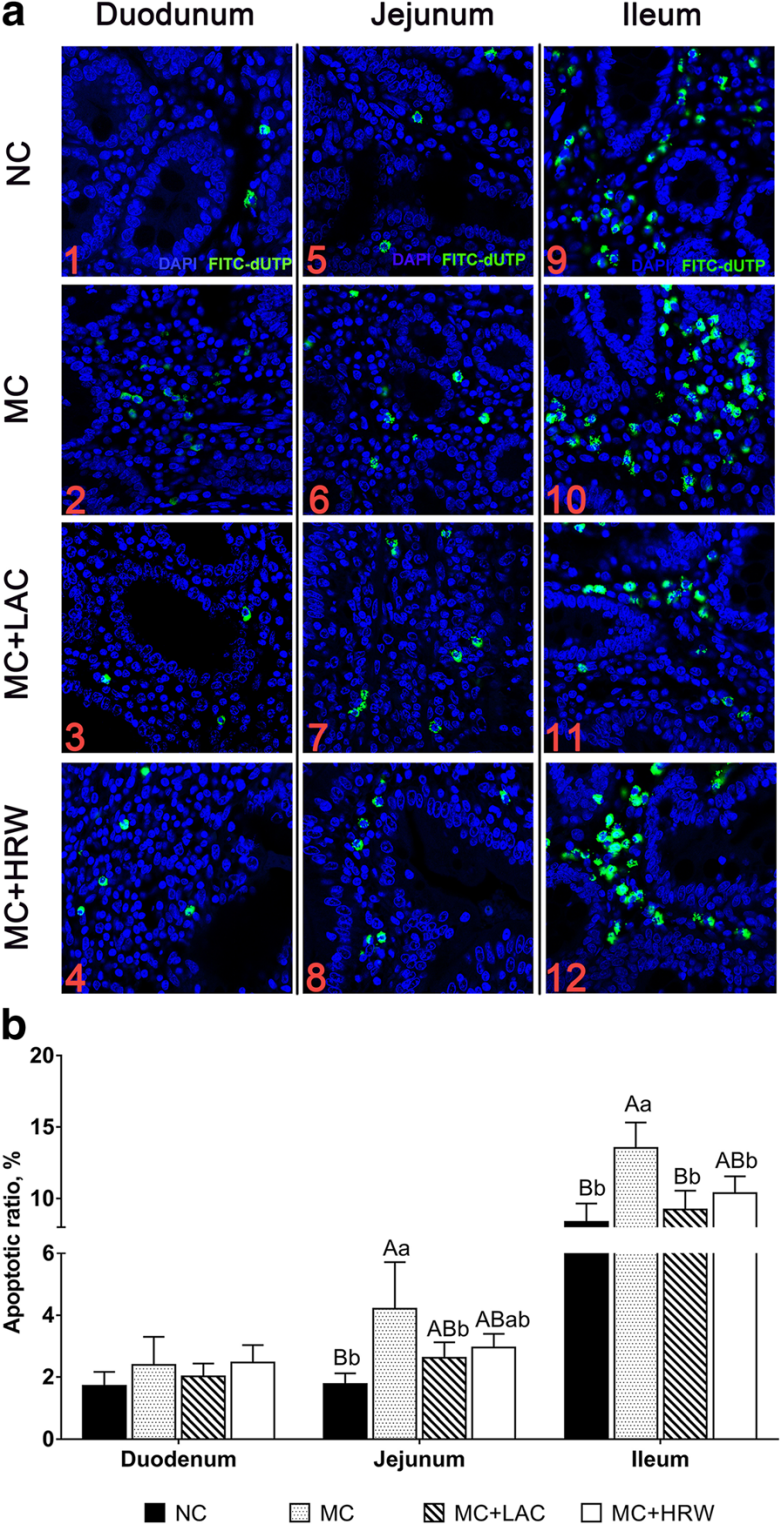
Effects of lactulose and hydrogen-rich water on the percentage of apoptosis intestinal epithelial cells by the TUNEL assay in female piglets fed *Fusarium* mycotoxin-contaminated diet. **a** Representative TUNEL stained paraffin sections from the duodenum (a1–4), jejunum (a5–8), and ileum (a9–12) tissue (original magnification, 400×). **b** Effects of lactulose and hydrogen-rich water on the percentage of small intestine apoptosis ratio in piglets fed *Fusarium* mycotoxin-contaminated diet. Each column represents the mean values (*n* = 5), with their standard deviation (SD) represented by vertical bars. Letters above the bars not sharing the same lower (*P* < 0.05) or upper (*P* < 0.01) case superscript letters are significantly different. NC = basal diet; MC = *Fusarium* mycotoxin-contaminated diet; MC + HRW = *Fusarium* mycotoxin-contaminated diet with hydrogen-rich water treatment; MC + LAC = *Fusarium* mycotoxin-contaminated diet with lactulose treatment

Representative observations of apoptosis in the duodenum (Fig. 3 a, a2, a3, and a4), jejunum (Fig. 3 a5, a6, a7, and a8), and ileum (Fig. 3a9, a10, a11, and a12) from NC, MC, MC + LAC, and MC + HRW groups were shown in Fig. 3a. Following in situ labeling of duodenum, jejunum, and ileum mucosal epithelium, stained epithelial cells from the jejunum and ileum were undergoing apoptosis in piglets fed MC diet were seen under microscopic examination. A number of TUNEL-positive cells were also found in piglets from MC + HRW and MC + LAC groups, respectively.

The apoptosis index for the quantification of TUNEL-positive cells is shown in Fig. 3b. In the duodenum, apoptotic index among the four groups was not different. In jejunum, an increase of apoptosis index was found in piglets fed MC diet than those that fed NC diet (*P* < 0.01). Compared with the MC group, only LAC treatment lowered the jejunum apoptosis index (*P* < 0.05). In ileum, MC group had a higher apoptosis index than any of NC, MC + LAC, and MC + HRW groups (*P* < 0.01). No difference in AI was seen among NC, MC + LAC and MC + HRW groups.

### mRNA changes of genes related to small intestinal barrier function

**Fig. 4:**
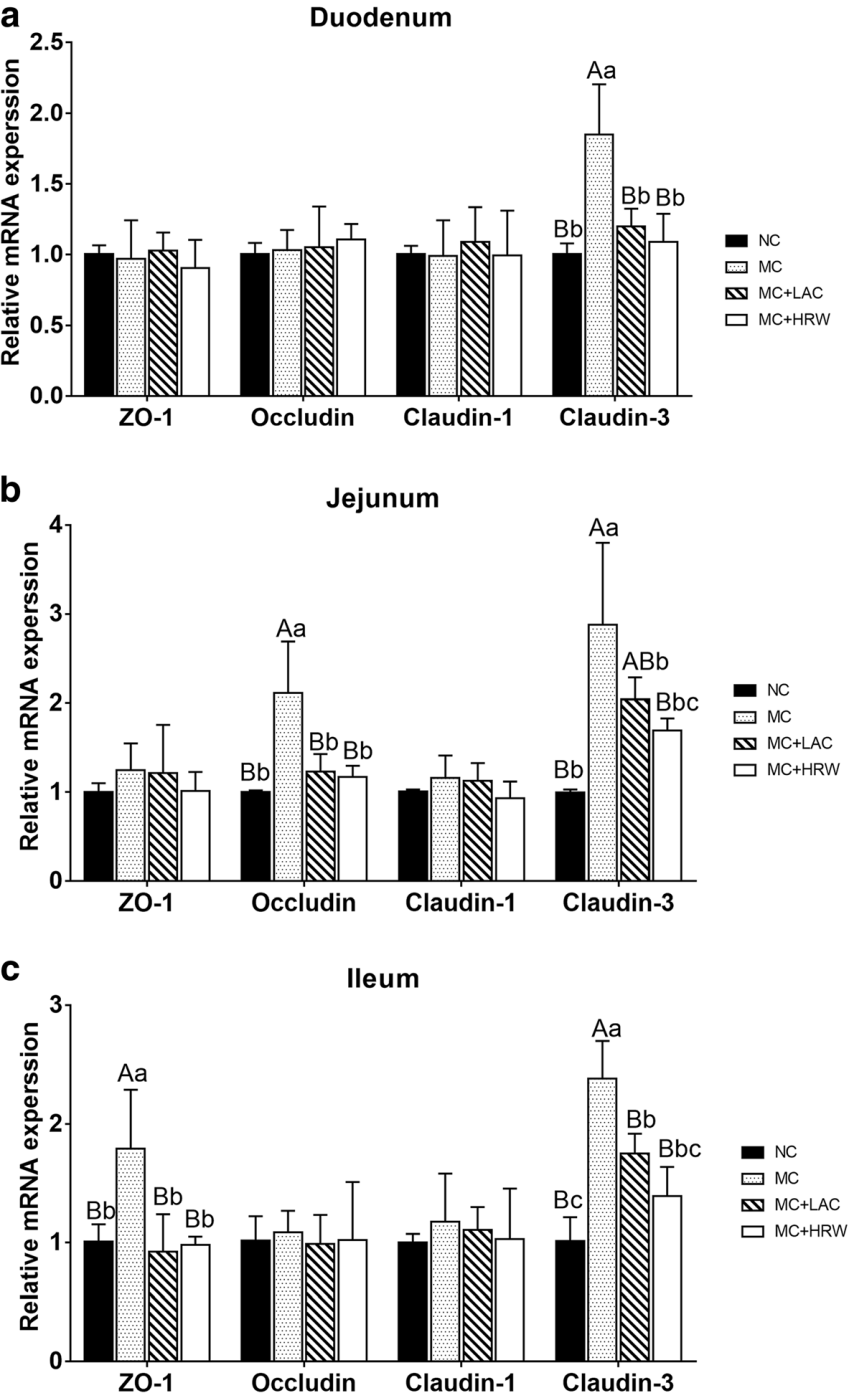
Effects of lactulose and hydrogen-rich water on relative mRNA gene expression levels related to tight junctions in the duodenum (**a**), jejunum (**b**), and ileum (**c**) of female piglets fed *Fusarium* mycotoxin-contaminated diet. Each column represents the mean values (*n* = 5), with their standard deviation (SD) represented by vertical bars. Letters above the bars not sharing the same lower (*P* < 0.05) or upper (*P* < 0.01) case superscript letters are significantly different. NC = basal diet; MC = mycotoxin-contaminated diet; MC + LAC = mycotoxin-contaminated diet with lactulose treatment. MC + HRW = mycotoxin-contaminated diet with hydrogen-rich water treatment

In duodenum (Fig. 4a), mRNA expression levels of *ZO-1*, *OCLN,* and *CLDN1* had no difference among the four treatment groups. mRNA expression levels of *CLDN3* in the MC group was higher than NC, MC + LAC, and MC + HRW groups (*P* < 0.01). No difference was detected among the NC, MC + LAC, and MC + HRW groups.

In jejunum (Fig. 4b), no difference was found in mRNA expression levels of *ZO-1* and *CLDN1* among the four treatment groups. MC diet stimulated the increase of *OCLN* and *CLDN3* mRNA expression levels compared with NC diet (*P* < 0.01). Both MC + LAC and MC + HRW groups had decreased *OCLN* and *CLDN3* mRNA expression levels compared with the MC group (*P* < 0.05).

In ileum (Fig. 4c), mRNA expression level of *ZO-1* in the MC group was higher than its expression level in NC, MC + HRW and MC + LAC groups (*P* < 0.01). No difference in *ZO-1* mRNA expression was seen among the MC, MC + LAC and MC + HRW groups. mRNA expression level of *CLDN3* in the MC group was higher than it in the NC, MC + LAC, and MC + HRW groups (*P* < 0.01). And *CLDN3* mRNA expression level in MC + LAC group was higher than the NC group (*P* < 0.05). However, mRNA expression levels of *OCLN* and *CLDN1* had no difference among the four treatment groups.

### Relative expression of CLDN3 protein in the small intestine

**Fig. 5:**
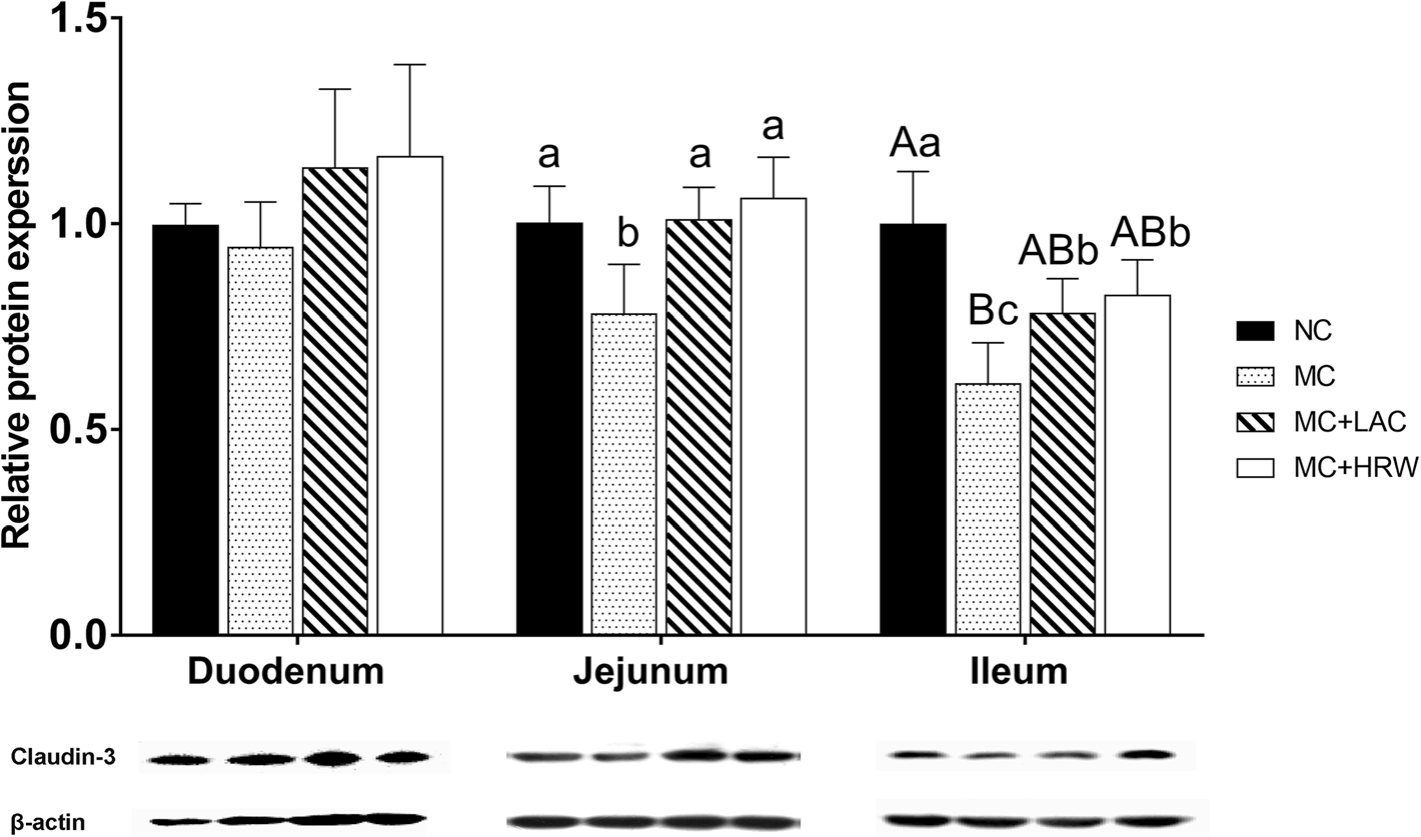
Effects of lactulose and hydrogen-rich water on claudin-3(CLDN3) protein expression in duodenum, jejunum, and ileum of female piglets fed *Fusarium* mycotoxin-contaminated diet. Each column represents the mean values (*n* = 5), with their standard deviation (SD) represented by vertical bars. Letters above the bars not sharing the same lower (*P* < 0.05) or upper (*P* < 0.01) case superscript letters are significantly different. NC = basal diet; MC = *Fusarium* mycotoxin-contaminated diet; MC + HRW = *Fusarium* mycotoxin-contaminated diet with hydrogen-rich water treatment; MC + LAC = *Fusarium* mycotoxin-contaminated diet with lactulose treatment

Using western blotting technique, no difference was found on the expression levels of CLDN3 protein in duodenum among the four treatment groups (Fig. 5). In both jejunum and ileum (Fig. 5), CLDN3 protein levels were lower in the MC group than in NC, MC + LAC, and MC + HRW groups (*P* < 0.05). No difference of CLDN3 protein expression level was detected among NC, MC + LAC, and MC + HRW groups in the jejunum (Fig. 5). While MC + LAC and MC + HRW groups had a higher ileum CLDN3 protein expression levels (*P* < 0.05) than MC group (Fig. 5).

### Distribution of CLDN3 protein in the surface of small intestinal

**Fig. 6:**
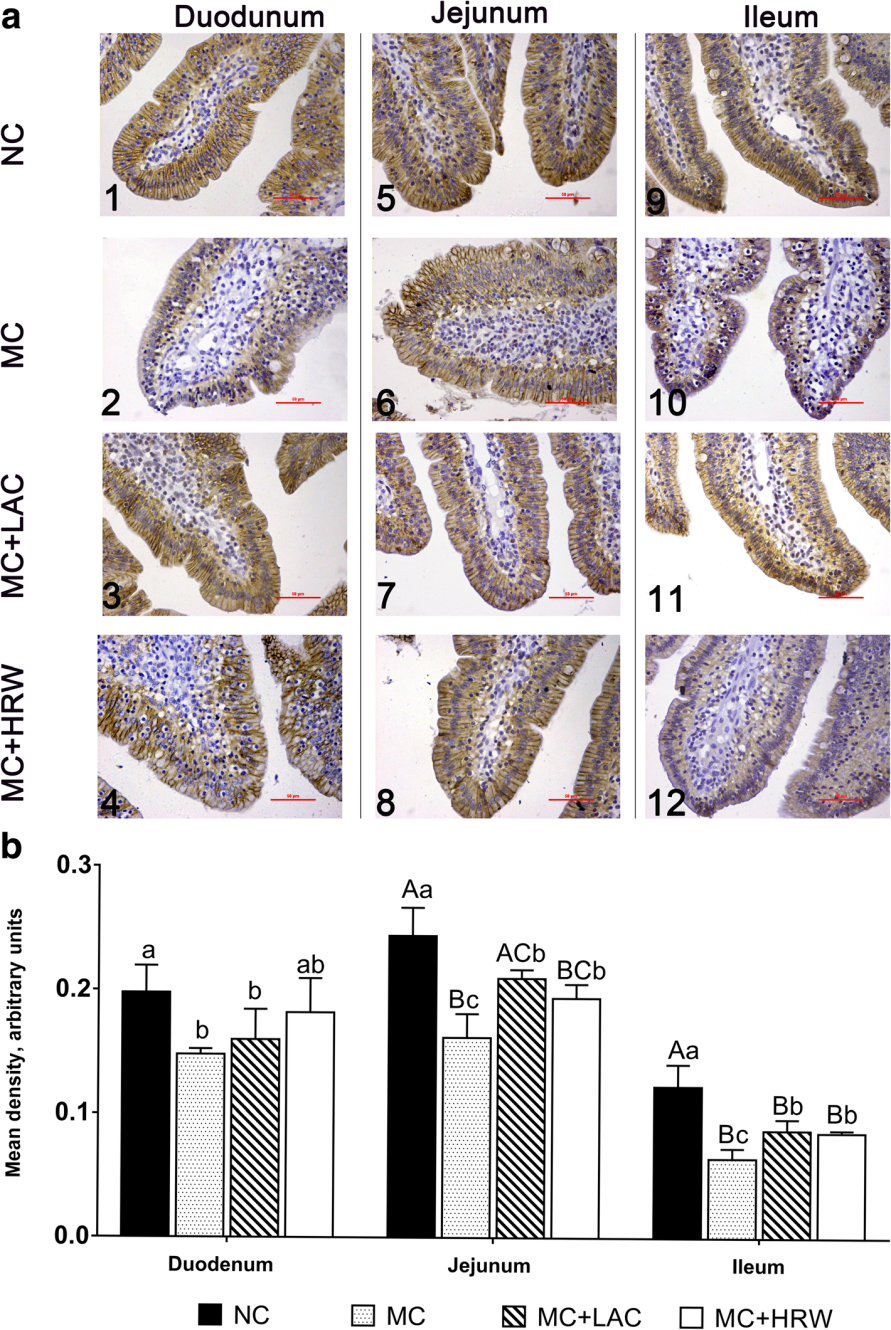
Effects of lactulose and hydrogen-rich water on claudin-3(CLDN3) protein density levels of the small intestine in female piglets fed *Fusarium* mycotoxin-contaminated diet. **a** Representative immunohistochemical staining images of CLDN3 protein in the duodenum (a1–4), jejunum (a5–8), and ileum (a9–12) were obtained at 400× magnification with red bar = 50 μm. **b** Mean density (arbitrary units) in the small intestine of piglets fed *Fusarium* mycotoxin-contaminated diet. Each column represents the mean values (*n* = 5), with their standard deviation (SD) represented by vertical bars. Letters above the bars not sharing the same lower (*P* < 0.05) or upper (*P* < 0.01) case superscript letters are significantly different. NC = basal diet; MC = *Fusarium* mycotoxin-contaminated diet; MC + HRW = *Fusarium* mycotoxin-contaminated diet with hydrogen-rich water treatment; MC + LAC = *Fusarium* mycotoxin-contaminated diet with lactulose treatment

The localization of CLDN3 protein status of the duodenum, jejunum, and ileum in four treatment groups was determined via immunohistochemistry assay. Strong positive immunoreactivity results were obtained in duodenum (Fig. 6 a1, a2, a3, and a4), jejunum (Fig. 6 a5, a6, a7, and a8), and ileum (Fig. 6 a9, a10, a11, and a12) indicated by brown staining (Fig. 6). CLDN3 protein can be seen on the full surface of villi especially clustered at the tips of villi (Fig. 6a). All three small intestinal villi in the NC group exhibited a continuous pattern of CLDN3 protein lining, which indicated good intestinal integrity (Fig. 6 a1, a5, and a9, respectively). *Fusarium* mycotoxin-contaminated diet was associated with a disturbed and irregular distribution of CLDN3 proteins in duodenum, jejunum, and ileum samples (Fig. 6 a2, a6, and a10), respectively. In the meanwhile, MC + HRW (Fig. 6 a4, a8, and a12) and MC + LAC (Fig. 6 a3, a7, and a11) groups had less irregular CLDN3 distribution than MC group.

The abundance of CLDN3 protein in the duodenum, jejunum, and ileum was also measured (Fig. 6b). Compared with NC group, MC group had a lower density of CLDN3 protein in duodenum, jejunum, and ileum (*P* < 0.05). In the duodenum, oral administration of either HRW or LAC did not alter CLDN3 protein expression density compared with the MC group. However, oral administrations of either HRW or LAC were found increased the CLDN3 expression levels when compared with the MC group in both jejunum and ileum (*P* < 0.05).

## Discussion

GIT is not only responsible for food ingestion, digestion, energy and nutrients absorption, but also an essential barrier preventing the passage of harmful intraluminal substances from the external environment [29]. Following the intake of *Fusarium* mycotoxin-contaminated feed, GIT can be exposed to high levels of toxic substances that consequentially damage intestinal functions [3]. HRW is known could ameliorate intestinal histologic damage and injury [14, 15]. LAC has also been shown protective effects against different models of intestinal damage through endogenous hydrogen [22–24]. Therefore, we hypothesized that oral administrations of either HRW or LAC could partially reverse the damages in small intestinal tracts caused by *Fusarium* mycotoxins in piglets.

The absorption of mycotoxins and their fate in the intestinal tract suggest that the gut epithelium is repeatedly exposed to these toxins, and at higher levels than other tissues [2–4]. Serum *D*-lactic acid levels, DAO activities, and endotoxin status are useful markers for measuring the permeability of the intestinal mucosa, intestinal injury and reperfusion insults [30]. In this study, *Fusarium* mycotoxin-contaminated feed induced abnormal intestinal tissue structural changes and altered intestinal permeability. A previous report [31] indicated that DON exposure not only caused a reduction in transepithelial electrical resistance (TEER) of intestinal epithelial cell monolayers but also increased the permeability of epithelial intestinal cell monolayers to bacteria. In vivo experiment also shown that serum concentrations of *D*-lactic acid and DAO were also elevated in piglets challenged with 4 mg/kg deoxynivalenol [32]. Those findings are consistent with our results that piglets fed *Fusarium* mycotoxin-contaminated feed had displayed higher serum *D*-lactic acid levels, DAO activities and LPS concentrations than those in the NC group.

It was reported that 2% hydrogen inhalation could attenuate I/R injury induced histopathological mucosal erosion and increased gut permeability via its antioxidant effects in rats [33]. Intra-peritoneal injection of hydrogen-rich saline (10 mL/kg) was also found to maintain the body weight, attenuate the severity of necrotizing enterocolitis (NEC), and prevent the increase of serum DAO in a neonatal rat model of NEC [34]. In this study, compared with the MC group, lower levels of serum *D*-lactic acid, DAO activities, and endotoxin concentrations were detected in both MC + LAC and MC + HRW groups. In our previous studies with exactly same piglets, oral administrations of HRW or LAC showed higher hydrogen concentrations in plasma and intestine [27, 28]. Although the underlying mechanisms of the HRW and LAC exert their protective effects on the gut permeability remains unknown, the antioxidative property of molecular hydrogen might shed the light on further discovering the underlying mechanism. Therefore, molecular hydrogen may be a good and novel candidate agent to reduce the side effects caused by *Fusarium* mycotoxins in piglets.

Changes of intestinal structure, including villus height, crypt depth and the ratio of villus height to crypt depth ratio are considered sensitive indicators of the intestine that reacts to the presence of harmful substances in feed [35]. Chronic (5-week long) ingestion of a diet contaminated with DON (3 mg/kg) alone or together with fumonisins (6 mg/kg) induced morphological changes in pig intestine, these changes included atrophy and fusion of villi, decreased of villi height, and cell proliferation in the jejunum [4]. In addition, feeding 5-week-old piglets for 28 d with multiple *Fusarium* toxin-contaminated feed caused decreased of villus height and crypt depth in both jejunum and ileum [36]. In our study, piglets fed *Fusarium* mycotoxin-contaminated diet had shorter villus height and a lower ratio of villus height to crypt depth in duodenum, jejunum, and ileum, suggesting that the physiological architecture of small intestine was compromised. These results might be partially accounted for higher serum DAO activities, *D*-lactic acids and LPS levels in the MC group than NC group due to increase intestinal permeability caused by small intestinal structure damage [31].

Furthermore, histological examination demonstrated that both HRW and LAC prevented *Fusarium* mycotoxin-induced mucosal structural changes in duodenum, jejunum, and ileum. A previous study reported that jugular venous cannula infusion of hydrogen-rich saline (5 mL/kg) significantly reduced the mucosa injury caused by IR, preventing shortened villi, loss of villous epithelium and prominent mucosa neutrophil infiltration in the small intestine of Sprague-Dawley rats [15]. Lactulose was able to reduce the colonic damage [24, 26] of DSS and trinitrobenzenesulfonic acid [25] models by increasing hydrogen production. So, it is not surprising to see that both HRW and LAC oral administrations have shown similar beneficial effects against *Fusarium* mycotoxin-induced intestinal damage in piglets. Since the intestinal is the key organ to digest feed and absorb nutrients, the protective effects of HRW and LAC on small intestinal morphology can be beneficial to improve growth performance in piglets [27].

Several studies indicated that mycotoxins belong to trichothecenes can cause apoptosis in bone marrow, marcophages, Peyer’s patches and thymus [37, 38]. Aflatoxin B1 (0.3 mg/kg) could induce the increase of apoptotic thymocyte by up-regulation mRNA expression level of *Bax* and caspase and down-regulation mRNA expression level of *Bcl-2* [39]. However, the effects of *Fusarium* mycotoxins on the apoptosis of small intestine were rarely explored in weaning piglets. In our study, feeding *Fusarium* mycotoxin-contaminated diet up-regulated *Bcl-2* and caspase-3 mRNA expression in jejunum, and caspase-3 expression in ileum. *Fusarium* mycotoxin-induced apoptosis is detected by TUNEL assay with higher epithelium apoptosis ratio in jejunum and ileum in MC group.

Molecular hydrogen has the ability to inhibit I/R-induced oxidative stress and apoptosis and promote epithelial cell proliferation [14, 17]. Hydrogen-rich saline could promote acinar cell proliferation, inhibit apoptosis and NF-κB activation from *L*-arginine-induced acute pancreatitis in rats [40]. In our study, 10 mL/kg BW of HRW (twice daily) and 500 mg/kg BW of LAC (twice daily) significantly down-regulated the apoptosis-related gene expression in the jejunum (*Bcl-2* and caspase-3) and ileum (caspase-3) compared with the MC group. The protective effects of HRW and LAC against *Fusarium* mycotoxins were also confirmed by TUNEL assay. Sun H et al., [41] has also reported a similar finding that the activation of caspase-3 decreased remarkably in the presence of hydrogen-rich saline.

It was also shown in our study that *CLDN3* mRNA expression was up-regulated by *Fusarium* mycotoxin-contaminated diet in the small intestine. Jejunum *OCLN* and ileum *ZO-1* mRNA expression levels were also up-regulated. Immunohistochemistry analysis results in small intestine sections also supported these results. These changes are also reported in other in vivo and in vitro studies. Up-regulation in mRNA expression levels of *CLDN3* and claudin-4 was observed in DON-exposed Caco-2 cells [42]. Low-dose (0.9 mg/kg feed), short-term exposure (10 d) of DON to piglets significantly changed the mRNA expression of different tight junction proteins in different parts of the small intestine [43]. However, no clear explanation for the contradicting results on mRNA, and protein expression levels of CLDN3. It can be speculated that this could be related to many factors such as exposure time, the age of piglets, and the compositions of *Fusarium* mycotoxins and the individual mycotoxin levels. Due to the replication limit, further experiments involved a large number of piglets and pure mycotoxins are definitely needed to be explored.

The association of excessive oxidative stress and *Fusarium* mycotoxin-induced intestinal barrier dysfunction has also been reported [10]. Our finding suggested that oral administrations of HRW and LAC not only attenuated the morphology damage of intestine but also protected the reduction of tight junctions in the small intestines caused by *Fusarium* mycotoxins. Previous studies in our lab demonstrated that endogenous hydrogen gas levels in the intestines and plasma were significantly improved by HRW and LAC [27, 28]. These endogenous gas may work against the side effects caused by *Fusarium* mycotoxins on tight junctions of the small intestine through its antioxidant and anti-inflammatory effects. H_2_ administration with different methods can contribute to prevention of severe intestinal diseases such as transplantation [33], ischemia/reperfusion injury [13, 17, 44] and colon inflammation [13, 14]. In addition, hydrogen-producing prebiotic (oligosaccharides and lactulose) has been demonstrated effective in intestinal inflammation models [24–26]. Furthermore, intestinal microbe also plays important roles in regulating the development and health of small intestine [28, 45]. Whether intestinal microbe plays a role in the beneficial effects of HRW and LAC in the current study remains unclear.

## Conclusions

In conclusion, this study demonstrated that oral administrations of HRW and LAC provided beneficial effects in reducing apoptosis of epithelium cells in small intestine, maintaining intestinal barrier, preventing intestinal morphological changes, and tight junctions disintegration, and restore the protein expression and distribution of CLDN3 in the small intestinal in female piglets fed *Fusarium* toxins contaminated diet. These findings provide a possible explanation for the curative effects of molecular hydrogen on *Fusarium* mycotoxins-induced growth depression, and a novel solution to alleviate the intestinal toxicity caused by *Fusarium* mycotoxins in swine production.

## Additional files

Additional file 1: **Table S1.** Ingredient composition and nutrient contents of control and experimental diets. (DOCX 20 kb)

Additional file 2: **Table S2.** List of primers used in this study. (DOCX 21 kb)

## Abbreviations

AI: Apoptosis index
BCA: Bicinchoninic acid
Bcl-2: B-cell CLL/lymphoma 2
BSA: Bovine serum albumin
BW: Body weight
CLDN1: Claudin-1
CLDN3: Claudin-3
DAO: Diamine oxidase
DON: Deoxynivalenol
DSS: Dextran sulfate sodium
EU: Endotoxin units
FAS: Fas cell surface death receptor
GIT: Gastrointestinal tract
H&E: Hematoxylin and eosin
HFW: Hydrogen-free water
HRW: Hydrogen-rich water
I/R: Ischemia-reperfusion
LAC: Lactulose
LPS: Lipopolysaccharide
MC: Mycotoxin-contaminated
NC: Negative control
OCLN: Occludin
PBS: Phosphate-buffered saline
SABC: Strept avidin-biotin complex
TUNEL: Terminal deoxynucleotidyl transferase dUTP nick end labeling
ZEN: Zearalenone
ZO-1: Zonula occludens 1
