# Supplementary material for: Morphological and molecular response of small intestine to lactulose and hydrogen-rich water in female piglets fed Fusarium mycotoxins contaminated diet
Source: J Anim Sci Biotechnol. 2019 Feb 13;10:9. doi: 10.1186/s40104-019-0320-2 (PMC6373143; doi:10.1186/s40104-019-0320-2)
Supplement: Supplementary file 1 — Table S1. Ingredient composition and nutrient contents of control and experimental diets. (DOCX 20 kb) [file 40104_2019_320_MOESM1_ESM.docx]

**Table S1.** Ingredient composition and nutrient contents of control and experimental diets

| Item | NC^1^ diet | MC^2^ diet |
| --- | --- | --- |
| Ingredients, % | | |
| Normal corn | 16.75 | 16.75 |
| *Fusarium* toxin uncontaminated corn | 44.50 | 0.00 |
| *Fusarium* toxin contaminated corn | 0.00 | 44.50 |
| Soybean meal | 15.79 | 15.79 |
| Extruded soybean | 10.00 | 10.00 |
| Fish meal | 5.00 | 5.00 |
| Wheat bran | 3.00 | 3.00 |
| Soybean oil | 1.74 | 1.74 |
| Vitamin and mineral premix^3^ | 1.00 | 1.00 |
| Limestone powder | 0.98 | 0.98 |
| Calcium hydrogen phosphate | 0.78 | 0.78 |
| Salt | 0.37 | 0.37 |
| Lysine HCl (98%) | 0.09 | 0.09 |
| Total | 100.00 | 100.00 |
| Analyzed chemical composition^4^ | | |
| DM, % | 88.96 | 88.28 |
| CP, % | 20.11 | 20.40 |
| Crude ash, % | 4.70 | 4.89 |
| Crude fiber, % | 1.71 | 1.96 |
| EE% | 8.04 | 8.65 |
| Calculated DE^5^, kcal/kg | 3400.00 | 3400.00 |

^1^NC, negative control (basal diet).

^2^MC, mycotoxin-contaminated diet.

^3^Provided, per kilogram of diet (as-fed basis) 55 mg Zn (ZnSO_4_), 30 mg Cu (CuSO_4_), 60 mg Mn (MnSO_4_), 120 mg Fe (FeSO_4_), 1 mg I (KI), 2 mg Co (CoSO_4_), 0.3 mg Se (Na_2_SeO_3_), 9000 IU vitamin A, 1800 IU vitamin D_3_, 40 IU vitamin E, 3 mg vitamin B_1_, 4.5 mg vitamin B_2_, 16 mg pantothenic acid, 10 mg vitamin B_6_, 0.08 mg vitamin B_12_, 28 mg niacin, 2 mg folic acid, 1.8 mg vitamin K_3_, 0.2 mg biotin, 800 mg choline chloride, and 100 mg vitamin C. The premix did not contain additional Cu, Zn, antibiotics, or probiotics.

^4^ Chemical composition are reported as fed basis unless indicated otherwise; CP, crude protein; EE, ether extract.^5^Based on a DM content of 88%.
