# Supplementary material for: Morphological and molecular response of small intestine to lactulose and hydrogen-rich water in female piglets fed Fusarium mycotoxins contaminated diet
Source: J Anim Sci Biotechnol. 2019 Feb 13;10:9. doi: 10.1186/s40104-019-0320-2 (PMC6373143; doi:10.1186/s40104-019-0320-2)
Supplement: Supplementary file 2 — Table S2. List of primers used in this study. (DOCX 21 kb) [file 40104_2019_320_MOESM2_ESM.docx]

**Table S2.** List of primers used in this study

| Gene^1^ | Primer sequences（5’→3’）^2^ | Size, bp | A_T_,^3^ °C | Reference |
| --- | --- | --- | --- | --- |
| *Bcl-2* | F: TCCAGAACCTCCTTGGTCCT | 187 | 60 | This study |
|  | R: AACTACAGCGAGGTGCTTCC |  |  |  |
| *Caspase-3* | F: GTGGGACTGAAGATGACA | 190 | 60 | [1] |
|  | R: ACCCGAGTAAGAATGTG |  |  |  |
| *FAS* | F: TGATGCCCAAGTGACTGACC | 103 | 60 | [1] |
|  | R: GCAGAATTGACCCTCACGAT |  |  |  |
| *ZO-1* | F:CAGAGACCAAGAGCCGTCC | 105 | 60 | [2] |
|  | R:TGCTTCAAGACATGGTTGGC |  |  |  |
| *OCLN* | F:AGGAGGTGGACTTTCAAGAGG | 118 | 60 | [2] |
|  | R: GCAGAATTGACCCTCACGAT |  |  |  |
| *CLDN1* | F: TTCTGGGAGGTGCCCTACTT | 74 | 60 | [3] |
|  | R: TGGATAGGGCCTTGGTGTTG |  |  |  |
| *CLDN3* | F:AACACCATCATCCGGGACTTC | 183 | 61.2 | [4] |
|  | R:CGCGGAGTAGAGGATCTTGG |  |  |  |
| *β-actin* | F:GGACTTCGAGCAGGAGATGG | 233 | 60 | [5] |
|  | R:GCACCGTGTTGGCGTAGAGG |  |  |  |

^1^ *Bcl-2*, B-cell CLL/lymphoma 2; *FAS*, Fas cell surface death receptor; *ZO-1*, zonula occludens 1; *OCLN*, occluding; *CLDN1*, claudin-1; *CLDN3*, claudin-3.

^2^ F=forward primer; R=reverse primer.

^3^ A_T_=annealing temperature.

**References**

1. Zhu L, Cai X, Guo Q, Chen X, Zhu S, Xu J. Effect of n-acetyl cysteine on enterocyte apoptosis and intracellular signalling pathways' response to oxidative stress in weaned piglets. Br J Nutr*.* 2013; 110: 1938-1947.10.1017/S0007114513001608.

2. Zhang Y, Zheng P, Yu B, He J, Yu J, Mao X B, et al. Dietary spray-dried chicken plasma improves intestinal barrier function and modulates immune status in weaning piglets. J Anim Sci*.* 2016; 94: 173-184.10.2527/jas.2015-9530.

3. Qi K K, Wu J, Deng B, Li Y M, Xu Z W. Pegylated porcine glucagon-like peptide-2 improved the intestinal digestive function and prevented inflammation of weaning piglets challenged with lps. Animal : an international journal of animal bioscience*.* 2015; 9: 1481-1489.10.1017/S1751731115000749.

4. Alizadeh A, Braber S, Akbari P, Garssen J, Fink-Gremmels J. Deoxynivalenol impairs weight gain and affects markers of gut health after low-dose, short-term exposure of growing pigs. Toxins (Basel)*.* 2015;7: 2071-2095.10.3390/toxins7062071.

5. Li G, Yao W, Jiang H. Short-chain fatty acids enhance adipocyte differentiation in the stromal vascular fraction of porcine adipose tissue. J Nutr*.* 2014; 144: 1887-1895.10.3945/jn.114.198531.
